# Technology-based therapy-response evaluation of axial motor symptoms under daily drug regimen of patients with Parkinson’s disease

**DOI:** 10.3389/fnagi.2022.901090

**Published:** 2022-08-05

**Authors:** Zhuang Wu, Ronghua Hong, Shuangfang Li, Kangwen Peng, Ao Lin, Yichen Gao, Yue Jin, Xiaoyun Su, Hongping Zhi, Qiang Guan, Lizhen Pan, Lingjing Jin

**Affiliations:** ^1^Neurotoxin Research Center of Key Laboratory of Spine and Spinal Cord Injury Repair and Regeneration of Ministry of Education, Department of Neurology, Tongji Hospital, School of Medicine, Tongji University, Shanghai, China; ^2^IFLYTEK Suzhou Research Institute, E4, Artificial Intelligence Industrial Park, Suzhou Industrial Park, Suzhou, China; ^3^Department of Neurology and Neurological Rehabilitation, Shanghai Yangzhi Rehabilitation Hospital, School of Medicine, Tongji University, Shanghai, China; ^4^Shanghai Clinical Research Center for Aging and Medicine, Shanghai, China

**Keywords:** Parkinson’s disease, depth camera, objective measurement, axial mobility, motor improvement

## Abstract

**Background:**

Axial disturbances are the most disabling symptoms of Parkinson’s disease (PD). Kinect-based objective measures could extract motion characteristics with high reliability and validity.

**Purpose:**

The present research aimed to quantify the therapy–response of axial motor symptoms to daily medication regimen and to explore the correlates of the improvement rate (IR) of axial motor symptoms based on a Kinect camera.

**Materials and methods:**

We enrolled 44 patients with PD and 21 healthy controls. All 65 participants performed the Movement Disorder Society-Sponsored Revision of the Unified Parkinson’s Disease Rating Scale part III and the Kinect-based kinematic evaluation to assess arising from a chair, gait, posture, and postural stability before and after medication. Spearman’s correlation analysis and multiple linear regression model were performed to explore the relationships between motor feature IR and clinical data.

**Results:**

All the features arising from a chair (*P* = 0.001), stride length (*P* = 0.001), velocity (*P* < 0.001), the height of foot lift (*P* < 0.001), and turning time (*P* = 0.001) improved significantly after a daily drug regimen in patients with PD. In addition, the anterior trunk flexion (lumbar level) exhibited significant improvement (*P* = 0.004). The IR of the axial motor symptoms score was significantly correlated with the IRs of kinematic features for gait velocity, stride length, foot lift height, and sitting speed (r_*s*_ = 0.345, *P* = 0.022; r_*s*_ = 0.382, *P* = 0.010; r_*s*_ = 0.314, *P* = 0.038; r_*s*_ = 0.518, *P* < 0.001, respectively). A multivariable regression analysis showed that the improvement in axial motor symptoms was associated with the IR of gait velocity only (β = 0.593, 95% CI = 0.023–1.164, *P* = 0.042).

**Conclusion:**

Axial symptoms were not completely drug-resistant, and some kinematic features can be improved after the daily medication regimen of patients with PD.

## Introduction

Parkinson’s disease (PD) is a common neurodegenerative disease characterized by tremors, rigidity, bradykinesia, and axial symptoms (Kalia and Lang, 2015). Axial symptoms, including gait and postural disorders, are among the most disabling symptoms which are responsible for progressive motor impairment and frequent falls in PD. Axial disturbances are saliently associated with non-motor symptoms, such as hypomimia (Ricciardi et al., 2020), anxiety (Sumec et al., 2017), and cognitive decline (Schneider et al., 2015; Pantall et al., 2018). In addition, PD patients with more severe axial symptoms are more likely to develop white matter hyperintensities (Lee et al., 2020; Jeong et al., 2021) and Pisa Syndrome (Liu et al., 2019). Axial motor impairments deeply reduce the quality of life in patients with PD (Cano-de-la-Cuerda et al., 2011; Bryant et al., 2016; Lau et al., 2019).

At present, the most commonly used method for the assessment of axial motor symptoms in PD is subjective reports of patients and the validated rating scales, such as the Movement Disorder Society-Sponsored Revision of the Unified PD Rating Scale part III (MDS-UPDRS III (Goetz et al., 2008). However, the assessment results are subjective and are easily biased by the experience of evaluators. In addition, treatment for patients with PD is symptomatic, which mainly relies on pharmacological treatment (Armstrong and Okun, 2020). Previous studies have focused on motor symptom improvement in patients with PD after monotherapy (Henderson et al., 2016; Smulders et al., 2016; Fabbri et al., 2019, 2020; Moreira et al., 2019) which is not in line with daily clinical practice. It is unlikely that a single treatment will be effective for all patients. Therefore, in the real world, most patients take several kinds of antiparkinsonian drugs at the same time, trying to obtain the greatest clinical benefits. Little is known about the therapy-response of axial motor symptoms under daily drug regimens in patients with PD. Accordingly, it is important to seek objective and reliable methods to evaluate patients’ axial motor symptoms and their responses to daily medication regimens.

With the rapid development of artificial intelligence, different technologies (such as RGB cameras, Kinect cameras, wearable sensors, and smartphones) have been used to measure the motor performance of patients with PD (Zhan et al., 2018; Wang et al., 2020; Di Lazzaro et al., 2021; Zhang et al., 2021). Of these technologies, the Kinect camera is a low-cost and powerful tool for extracting motion characteristics with high reliability and validity (Clark et al., 2019).

Given this background, the present research aimed to quantify the therapy–response of axial motor symptoms to daily medication regimen and to explore the relationships between the improvement of axial motor symptom score and the changes in kinematic features. In-depth knowledge of specific axial motor symptom impairments and improvements after patients’ daily drug regimen will aid in patient-tailored treatment and lead to more effective management strategies for PD.

## Materials and methods

### Participants

Twenty-one healthy control (HC) participants and 44 patients with PD were recruited from the Department of Neurology, Tongji Hospital of Tongji University between March 2021 and February 2022. Inclusion criteria for HC were as follows: (1) no medical history of PD, stroke, spinal column diseases, and orthopedic disease; (2) ability to understand and follow doctor’s instructions. Inclusion criteria for PD were as follows: (1) diagnosis of idiopathic PD according to Movement Disorder Society (MDS) clinical diagnostic criteria (Postuma et al., 2015); (2) no medical history of stroke, spinal column diseases, and orthopedic disease; (3) taking the anti-PD medicine stably according to their daily drug regimen for at least 1 month; and (4) can understand and follow the doctor’s instructions. Demographic and clinical data were collected, including age, height, weight, body mass index (BMI), gender, education level, the combination of antiparkinsonian drugs, levodopa equivalent dose (LED), and disease duration. For all patients with PD, they had stopped antiparkinsonian drugs for at least 24 h and this time was defined as an OFF state. At the same time, the first MDS-UPDRS III score and motor features were collected. Approximately an hour later, the second MDS-UPDRS III score and motor features were collected again when patients with PD felt the best response to their daily medication regimen. As previous studies reported, the axial symptom score consisted of the following seven sub-items such as 3.1 speech; 3.3 neck stiffness; 3.9 arising from the chair; 3.10 gait; 3.11 freezing of gait; 3.12 postural stability, and 3.13 posture (Fabbri et al., 2019; Lau et al., 2019; Ricciardi et al., 2020). The improvement rate (IR) of axial motor symptom score and kinematic features were calculated as follows:


$$\%\text{IR}=\frac{\left(X_{\text{OFF}}-X_{\text{ON}}\right)}{X_{\text{OFF}}}\times 100$$

*X* indicated the MDS-UPDRS III score, axial motor symptom score and the kinematic features.

The Ethical Committee of Tongji Hospital approved the study (IRB No. 2019-061). All participants provided written informed consent before the research. All the mentioned procedures were performed according to the Declaration of Helsinki.

### Device

The motion analysis device consisted of an Azure Kinect depth camera (depth camera 1024 × 1024 pixels @30fps, 7-microphone linear phased array, RGB 3840 × 2160 pixels @30fps, Microsoft), a lateral RGB camera (MCD-400W plane, Ming Chuangda), a frontal RGB camera (MCD-400W plane, Ming Chuangda), a guide screen, and an independent computer. A complete set of algorithms were developed by the iFLYTEK Suzhou Research Institute to acquire the kinematic features.

### Axial motor features

The following axial motor features were included in the analysis.

#### Arising from a chair

Each participant sat in the chair comfortably and then was instructed to cross their arms across their chest and to stand up from the chair. The following features were collected: sitting time and sitting speed; and rising time and rising speed.

#### Gait

All participants stood quietly with their arms at their sides. Then, they walked for 3 m in a self-selected and comfortable way, turned 180°, and returned to their initial place. They walked back and forth three times and finally returned to the starting point. The following gait features were collected: stride length, velocity, cadence, stride time, double stance phase time (StPT), single StPT, swing phase time (SwPT), height of foot lift, step width, and turning time.

#### Postural stability

Each participant opened their eyes and stood erect with their feet comfortably apart. The doctor stood behind the participant and pulled the participant forcefully and briskly two times. The average number of retropulsive steps of participants was calculated.

#### Posture

**FIGURE 1:**
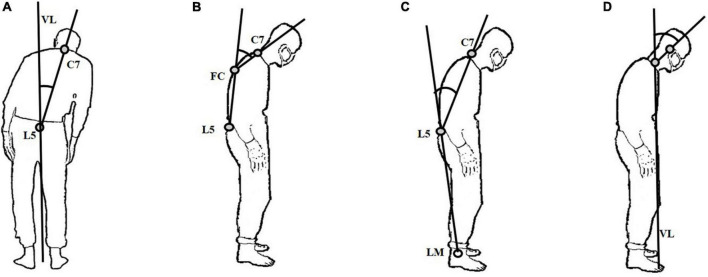
Illustration of the measured features for abnormal postures. **(A)** lateral trunk flexion; **(B)** anterior trunk flexion (thoracic level); **(C)** anterior trunk flexion (lumbar level); **(D)** anterior neck flexion.

**FIGURE 2:**
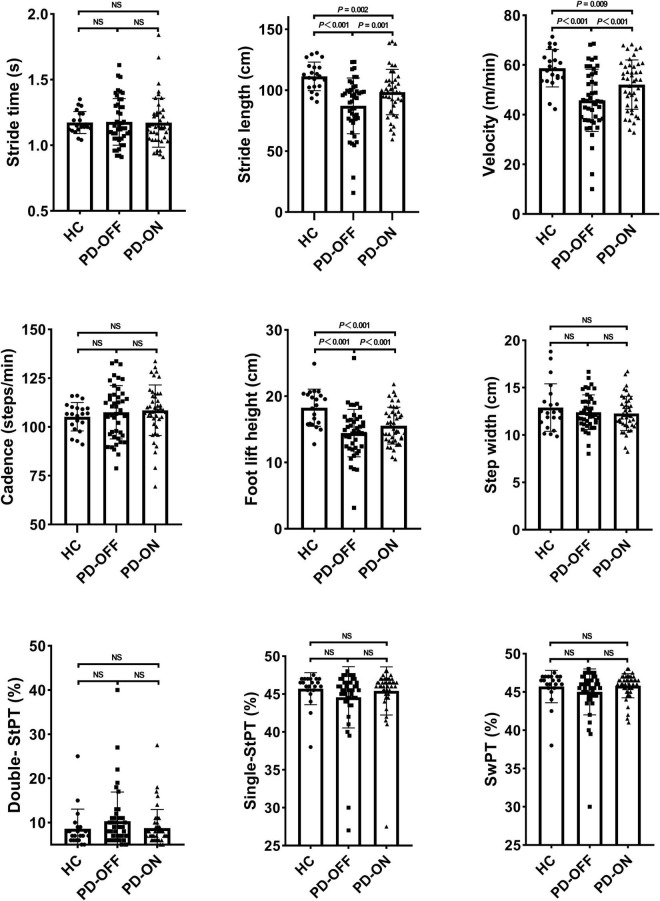
Differences in gait parameters. StPT, stance phase time; SwPT, swing phase time; NS, no significance.

**FIGURE 3:**
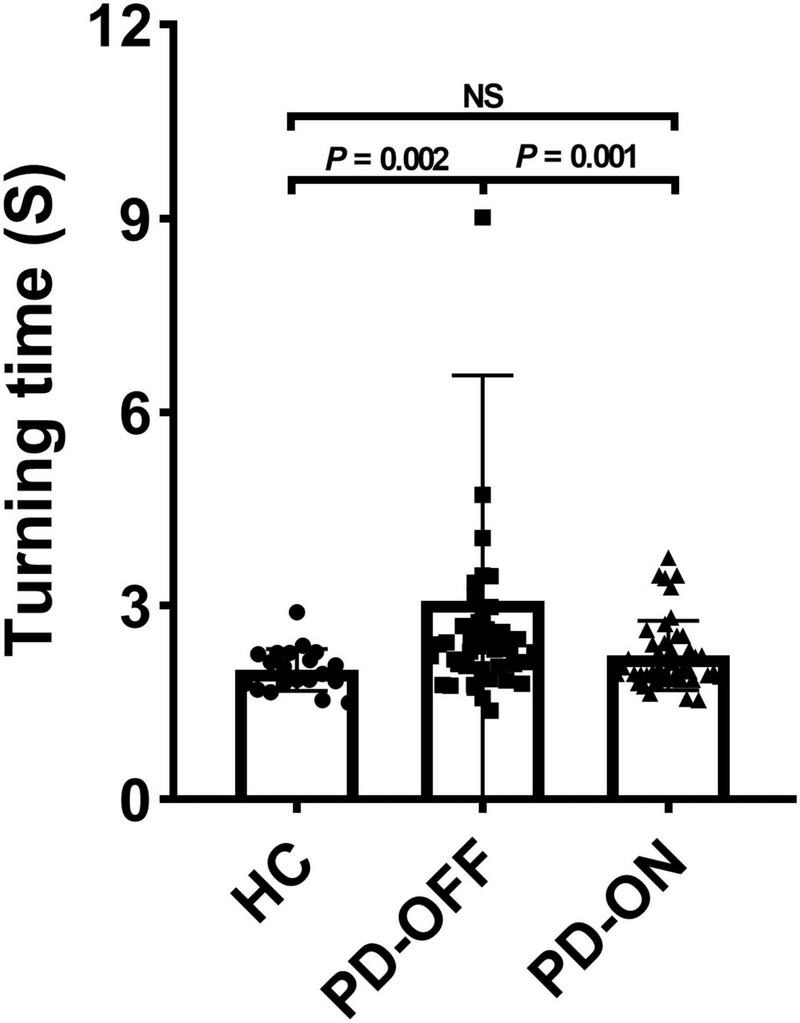
Differences in the turning time of participants.

**FIGURE 4:**
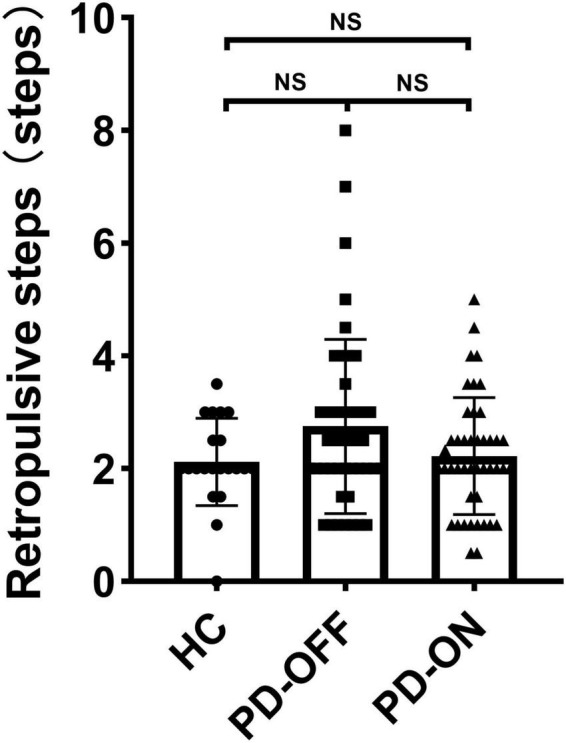
Number of retropulsive steps of participants.

To observe the flexion and side-to-side leaning of participants, they were instructed to stand with their backs to the Kinect depth camera. Subsequently, we asked them to turn right at 90°. According to the latest consensus released by the International Parkinson and Movement Disorders Society Task Force on Postural Abnormalities and previous studies (Ando et al., 2019; Geroin et al., 2020; Zhang et al., 2021; Tinazzi et al., 2022), the following four posture features, namely, lateral trunk flexion, anterior trunk flexion (thoracic level), anterior trunk flexion (lumbar level), and anterior neck flexion, were collected: (1) lateral trunk flexion. Connect the spinous processes of the 7th cervical vertebra (C7) and the 5th lumbar vertebra (L5) in the coronal plane. The angle between the line and the vertical line of the ground (VL) is the lateral trunk flexion angle (Figure 1A); (2) anterior trunk flexion (thoracic level). Connect L5 and the most convex point of the vertebra (FC). Then, connect FC and C7. The angle between the above two lines on the sagittal plane is defined as anterior trunk flexion (thoracic level) (Figure 1B); (3) anterior trunk flexion (lumbar level). Firstly, connect the L5 and lateral malleolus (LM). Then, connect the L5 and C7. The angle between the two lines on the sagittal plane is defined as anterior trunk flexion (lumbar level) (Figure 1C); (4) anterior neck flexion, the angle between the connecting line of the midpoint of the neck and the external acoustic foramen on the sagittal plane and VL is defined as anterior neck flexion (Figure 1D).

### Statistical analysis

The normality of the distribution of quantitative data was initially tested using the Shapiro–Wilk test. Quantitative data were presented as mean ± SD or median (interquartile range) as appropriate. Categorical variables were shown as frequency and proportion and were tested using the chi-square test. To compare the corresponding difference of the related data among the HC, PD-OFF, and PD-ON groups, a one-way analysis of variance or the Kruskal–Wallis *H* test was used. Then, a paired *t*-test or an independent *t*-test was adopted to compare the differences between the two groups if both sets of data followed a normal distribution. For non-normally distributed related data, the Wilcoxon signed-rank test or the Mann–Whitney *U*-test was used. To compare the effects of different treatment options on axial motor symptoms, we used LED as a covariate when we made comparisons. Spearman’s correlation analysis was performed to explore the relationships between motor feature IR and clinical data. Then, the variables that were significantly correlated with the IR of axial motor symptom score at the univariable level were then included in the multivariable linear regression model. For all analyses, the significance level was set to a *p*-value of <0.05. At the same time, because of multiple comparisons among the HC, PD-OFF, and PD-ON groups, the Bonferroni method was used. The alpha value was set at *P*′ = 0.05/times of comparison and that is *P*′ = 0.017. All statistical procedures were performed using SPSS software version 25 (IBM Corp, Armonk, NY, United States). GraphPad Prism version 8.0.1 was used in the figure configuration.

## Results

### Clinical characteristics of participants

Demographical and clinical data of 65 participants enrolled in the study were presented in Table 1. There was no difference in all baseline data between the two groups. For the PD group, the mean axial symptom scores were 5.39 ± 2.73 and 3.57 ± 2.03, respectively. The IR of the axial motor symptom score was 31.55 ± 29.72%.

**TABLE 1:** Clinical characteristics of participants.

	HC	PD	*P*
N	21	44	
Age	68.00 (65.00, 73.00)	68.00 (64.00, 70.00)	0.372
Height	164.00 ± 7.10	164.66 ± 8.11	0.751
Weight	62.98 ± 13.30	61.41 ± 9.18	0.581
BMI	23.26 ± 3.80	22.62 ± 2.80	0.444
Male (n, %)	10 (47.62)	24 (54.55)	0.791
Education (n, %)			0.803
Primary school	0 (0.00)	1 (2.27)	
Middle school	6 (28.57)	11 (25.00)	
High school	9 (42.86)	15 (34.09)	
College	6 (28.57)	17 (38.64)	
Morning LED (mg)	NA	185.42 ± 89.68	NA
LEDD (mg)	NA	470.36 ± 172.44	NA
Duration of PD (years)	NA	7.20 ± 3.45	NA
**OFF state**			
H-Y stage	NA	2.00 (2.00, 2.50)	NA
MDS-UPDRS III score	NA	34.70 ± 12.50	NA
Axial symptom score	NA	5.39 ± 2.73	NA
**ON state**			
H-Y stage	NA	2.00 (2.00, 2.00)	NA
MDS-UPDRS III score	NA	25.91 ± 12.39	NA
Axial symptom score	NA	3.57 ± 2.03	NA
MDS-UPDRS III improvement rate (%)	NA	26.34 ± 18.26	NA
IR of axial motor symptom score (%)	NA	31.55 ± 29.72	NA

BMI, body mass index; LED, levodopa equivalent dose; LEDD, levodopa equivalent daily dose; H-Y stage, Hoehn-Yahr stage; MDS-UPDRS III, Movement Disorder Society sponsored revision of the Unified Parkinson’s Disease Rating Scale part III; IR, improvement rate; NA, not applicable.

### Axial kinematic features

#### Arising from a chair

As reported in Table 2, we found a significant difference in all features (*P* < 0.05). Compared with the HC group, *post hoc* analysis revealed an increase in rising time (*P* < 0.001) and sitting time (*P* < 0.001); a decrease in rising speed (*P* = 0.009) and sitting speed (*P* < 0.001) in the PD-OFF group. Compared to the PD-OFF group, there were significant improvements in all features (*P* = 0.001) in the PD-ON group. Notably, no difference was observed between the HC and PD-ON groups in rising speed (*P* = 0.118).

**TABLE 2:** Differences in kinematic features of arising from a chair.

	HC	PD-OFF	PD-ON	*P*	Post hoc tests
Rising time (s)	0.57 ± 0.09	1.04 ± 1.90	0.69 ± 0.16	**<0.001**	**<0.001^a^**,** <0.001^b^**, **0.001^c^**
Rising speed (cm/s)	43.73 ± 6.92	37.63 ± 9.25	40.54 ± 7.88	**0.034**	**0.009^a^, 0.001^c^**
Sitting time (s)	0.58 ± 0.05	1.04 ± 1.90	0.69 ± 0.16	**<0.001**	**<0.001^a^**,** <0.001^b^**, **0.001^c^**
Sitting speed (cm/s)	39.70 ± 6.30	31.31 ± 7.53	34.17 ± 7.11	**<0.001**	**<0.001^a^**, **0.003^b^**, **0.001^c^**

Bold font means significant results. ^a^Comparison between the HC and PD-OFF groups. ^b^Comparison between the HC and PD-ON groups. ^c^Comparison between PD-OFF and PD-ON group.

#### Gait

We found a significant difference in stride length (*P* < 0.001), velocity (*P* < 0.001), the height of foot lift (*P* < 0.001; Figure 2), and turning time (*P* = 0.005; Figure 3). Compared with the PD-OFF group, there were significant improvements in stride length (*P* = 0.001), velocity (*P* < 0.001), and the height of foot lift (*P* < 0.001) in the PD-ON group. For turning time, there was no difference between the HC and PD-ON groups (*P* = 0.124).

#### Postural stability

As shown in Figure 4, there was no difference among the participants for postural stability (*P* = 0.317).

#### Posture

As reported in Table 3, we found a significant difference in anterior trunk flexion (lumbar level) (*P* < 0.001) and anterior neck flexion angle (*P* = 0.019). Compared with the HC group, *post hoc* analysis found an increase in anterior trunk flexion (lumbar level) (*P* < 0.001) and anterior neck flexion angle (*P* = 0.007) in the PD-OFF group. Compared with the PD-OFF group, there was a significant improvement in anterior trunk flexion (lumbar level) (*P* = 0.004) in the PD-ON group. There were trends for improvements in anterior neck flexion angle (*P* = 0.058) between the PD-OFF and PD-ON groups.

**TABLE 3:** Differences in postural features of participants.

	HC	PD-OFF	PD-ON	*P*	Post hoc tests
LTF (°)	1.08 (1.03, 2.03)	1.41 (0.24, 2.19)	1.03 (0.92, 2.04)	0.707	NA
ATF (thoracic level) (°)	28.61 (17.38, 34.19)	32.39 (27.87, 39.04)	31.88 (24.19, 37.56)	0.201	NA
ATF (lumbar level) (°)	12.18 (7.17, 13.61)	16.30 (13.29, 19.37)	14.41 (10.33, 17.15)	**<0.001**	**<0.001^a^**, **0.006^b^**, **0.004^c^**
ANF (°)	20.56 (12.99, 36.87)	36.87 (18.33, 36.87)	30.96 (16.70, 36.87)	**0.019**	**0.007^a^**

Bold font means significant results. ^a^Comparison between the HC and PD-OFF groups. ^b^Comparison between the HC and PD-ON groups. ^c^Comparison between PD-OFF and PD-ON group. LTF, lateral trunk flexion; ATF, anterior trunk flexion; ANF, anterior neck flexion.

#### Changes in kinematic features of participants after taking the medicine

**FIGURE 5:**
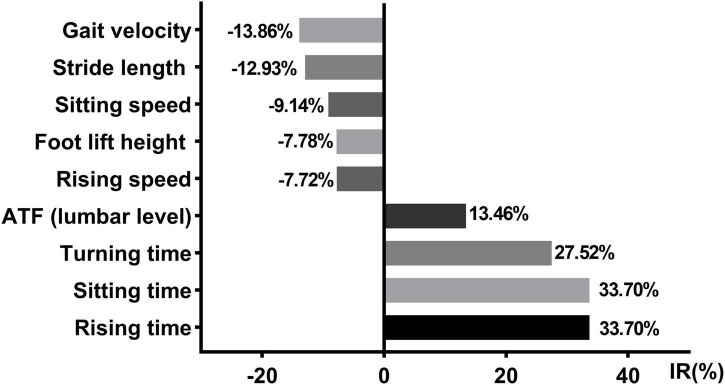
Axial kinematic features changed with the daily medication program. IR, improvement rate; ATF, anterior trunk flexion; Data were shown in %.

We observed significant improvements in gait velocity, stride length, foot lift height, turning time, rising speed, rising time, sitting speed, sitting time, and anterior trunk flexion (lumbar level) in the PD group. Then, we calculated the rate of change for these features (Figure 5).

#### Comparisons of kinematic features improvement rates among different treatment groups

To explore the possible influence of different treatment options on the improvement of axial symptoms, we calculated and compared the improvement rates of these kinematic features. Of the 44 patients with PD, nine patients were treated with levodopa only; 15 patients were treated with levodopa and dopamine agonists (DA); and seven patients were treated with the combination of levodopa, DA, and monoamine oxidase type B inhibitors (MAOB-I) (For the combinations of anti-PD drugs in all patients, please see Supplementary Table 1). In our research, the improvement rates of the axial motor features and LED were calculated. When controlling for LED, no difference was observed among the three groups (Table 4).

**TABLE 4:** Comparisons of kinematic feature improvement rates among three groups after controlling for LED.

	Levodopa (N = 9; LED = 111.11 ± 22.05)	Levodopa + DA (N = 15; LED = 142.50 ± 49.28)	Levodopa + DA + MAOB-I (N = 7; LED = 242.26 ± 42.11)	*P* (controlling for LED)
IR of axial motor symptom score	31.34 ± 22.30	26.00 ± 19.55	45.48 ± 26.64	0.566
Gait velocity IR	−13.51 (−18.82, 1.57)	−12.30 (−25.46, −5.06)	−15.68 (−25.61, 11.45)	0.089
Stride length IR	−2.29 (−17.30, 8.87)	−10.98 (−21.40, −2.03)	−7.10 (−24.71, 7.50)	0.289
Foot lift height IR	−4.50 (−16.20, 5.11)	−9.52 (−11.90, −3.58)	−7.42 (−9.18, 14.88)	0.177
Turning time IR	16.22 (2.61, 19.25)	4.36 (−8.15, 26.48)	2.31 (−7.91, 27.51)	0.334
Rising speed IR	−16.56 (−28.67, −5.34)	−3.93 (−6.85, 1.87)	−3.74 (−12.03, 1.52)	0.649
Rising time IR	19.78 (7.22, 30.91)	1.56 (0, 8.20)	10.42 (−8.96, 16.46)	0.159
Sitting speed IR	−12.11 (−20.22, −1.05)	−4.63 (−12.89, −0.38)	−11.29 (−29.56, −4.46)	0.980
Sitting time IR	19.78 (7.22, 30.91)	1.56 (0, 8.20)	10.42 (−8.96, 16.46)	0.159
ATF (lumbar level) IR	14.90 (−5.64, 31.34)	19.63 (−7.99, 26.00)	3.28 (−12.32, 27.40)	0.251

Data were shown as median (interquartile range). LED, levodopa equivalent dose; IR, improvement rate; DA, dopamine agonist; MAOB-I, monoamine oxidase type B inhibitors; ATF, anterior trunk flexion.

#### Correlation coefficients for clinical data and improvement of kinematic features in the Parkinson’s disease group

We explored the correlation coefficients for clinical data and IR of some kinematic features in the PD group (Table 5). The IR of the axial motor symptoms score was significantly correlated with the IRs of kinematic features for gait velocity, stride length, foot lift height, and sitting speed (respectively, r_*s*_ = 0.345, *P* = 0.022; r_*s*_ = 0.382, *P* = 0.010; r_*s*_ = 0.314, *P* = 0.038; r_*s*_ = 0.518, *P* < 0.001). Multivariable regression analysis showed that the improvement in axial motor symptoms was associated with the IR of gait velocity (β = 0.593, 95% CI = 0.023–1.164, *P* = 0.042; Table 6).

**TABLE 5:** Correlation coefficients for clinical data and improvement of kinematic features in Parkinson’s disease (PD) group.

	Age	LED	Disease duration	MDS-UPDRS III IR	Axial symptom score IR
ATF (lumbar level) IR	−0.241	0.143	0.020	0.189	0.264
Gait velocity IR	0.050	0.259	0.189	0.294	**0.345** *
Stride length IR	−0.010	0.242	0.012	0.107	**0.382** *
Foot lift height IR	−0.014	0.109	0.117	0.256	**0.314** *
Turning time IR	−0.113	0.074	0.164	0.228	0.218
Rising speed IR	0.225	−0.131	0.054	−0.023	0.278
Rising time IR	−0.013	0.021	0.126	0.165	0.296
Sitting speed IR	−0.087	0.100	0.180	0.245	**0.518** **
Sitting time IR	−0.013	0.021	0.126	0.165	0.296

Bold font means significant results. *P < 0.05, **P ≤ 0.001. ATF, anterior trunk flexion. IR, improvement rate. MDS-UPDRS III, Movement Disorder Society sponsored revision of the Unified Parkinson’s Disease Rating Scale part III.

**TABLE 6:** Multiple linear regression analysis with improvement rate of axial motor symptom score as a dependent variable in the Parkinson’s disease (PD) group.

	β	Std. error	Standard β	*P*	95% CI, lower bound	95% CI, upper bound
Gait velocity IR	0.593	0.282	0.991	**0.042**	0.023	1.164
Stride length IR	−0.336	0.250	−0.822	0.186	−0.842	0.169
Foot lift height IR	0.068	0.318	0.094	0.831	−0.575	0.712
Sitting speed IR	0.214	0.183	0.178	0.251	−0.157	0.584

Bold font means significant results; IR, improvement rate; CI, confidence interval.

## Discussion

In this study, we developed a whole set of intelligent evaluation systems to assess the axial symptoms of patients with PD and to evaluate their response to daily medication regimes using a Kinect camera. Our results demonstrated that axial symptoms were not completely drug-resistant. All the features arising from a chair, some gait and posture features can be improved after a daily drug regimen. In addition, the IRs of these motor features with medication were associated with the IR of axial motor symptom score. Our research provided an objective and reliable system to evaluate axial motor symptoms in patients with PD.

An ability to rise from a chair is significant for maintaining independent living and it is associated with the life quality of patients with PD (Bryant et al., 2020). Our results revealed both the rising speed and rising time were impaired in patients with PD, which is in agreement with previous studies (Inkster et al., 2003). Furthermore, our research showed that the ability of patients with PD to rise from a chair can be improved with their daily medication regimen. Compared with the OFF state, the rising speed and sitting speed increased remarkably by ∼7.72 and ∼9.14% in the ON state of patients with PD, respectively. Rising time and sitting time decreased significantly by ∼33.70 and 33.70% in the ON state compared to that of the OFF state in patients with PD, respectively. However, these are not complete improvements, and there is still a difference between the PD-ON group and healthy control subjects. Previous studies demonstrated the bad performance of rising from a chair in patients with PD can be attributed to their insufficient lower extremity strength, particularly at the hip (Bean et al., 2002; Inkster et al., 2003). Our findings indicate a rehabilitation method that focuses on the lower extremities is a necessary and preferred rehabilitation strategy in patients with PD.

As to gait performance, it has been well-known that patients with PD exhibited decreased stride length, slow turns, reduced velocity, and a small height of foot lift (Gavriliuc et al., 2020; Morrison et al., 2021; Wu et al., 2021; Zanardi et al., 2021). Similar results were also revealed in our research. Our research revealed that the improvements in gait performance of patients with PD are usually insufficient when compared to healthy control subjects. There is still a gap between the PD-ON group and the HC group in some gait features. At present, rehabilitation therapy is gaining more and more attention to aid in PD treatment. Similar to “bradykinesia,” “shuffling” is also an important feature of gait in patients with PD. Our research revealed that foot lift height remarkably decreased in the PD group. Decreased foot lift height in the swing phase indicated that patients with PD had worse foot clearance ability, which is highly associated with falls (Alcock et al., 2018). Accordingly, rehabilitation therapy, as a complementary treatment to antiparkinsonian drugs, those approaches that focus on increasing toe-ground clearance should be the preferred option. These rehabilitation programs include, for example, an attentional strategy emphasizing heel strike (Ginis et al., 2017) and biofeedback gait training measures (Nagano et al., 2020; Tiwari and Joshi, 2021).

Another relevant common complication of PD is postural abnormalities. For the time being, the effect of pharmacological treatment on posture abnormities is controversial. Some studies found that treatment with levodopa improved posture abnormalities, while others found little or no improvement (Ponfick et al., 2011; Barone et al., 2016). However, little is known about the therapy response to daily medication programs of patients with PD. Our research extended previous studies and demonstrated anterior trunk flexion (lumbar level) angle exhibited improvements after taking medicine in patients with PD. Notably, the improvement rate of this angle was 13.46%, with its median value dropping from 16.30° in the OFF state to 14.41° in the ON state. According to the latest consensus released by the International Parkinson and Movement Disorders Society Task Force on Postural Abnormalities (Tinazzi et al., 2022), 15° of anterior trunk flexion is the boundary between normal posture and milder postural abnormalities. This means that some postural parameters can be improved with their daily medication regimen from a clinical standpoint. However, we did not observe a difference in other posture measures. A previous study reported there were two different phenotypes of levodopa-responsiveness in PD patients with posture abnormities (Kataoka and Ueno, 2017). Patients with lateral trunk flexion poorly respond to levodopa, while the angle of the anterior trunk flexion significantly decreased after the infusion of levodopa. These findings indicated that posture abnormities of patients with PD can gain some benefits from their intake of medicine, but the response of axial symptoms to medical therapy cannot be generalized. The reason behind this may be related to the varying severity of the patient’s posture abnormities and the involvement of different complexity of the affected muscles in different kinds of abnormal postures.

Another relevant finding of the present study was that a reduction in axial motor symptoms is associated with the IRs of some kinematic features. It indicated that the extent of improvements in these motor features can be used to reflect the overall changes in axial symptoms. This set of a system can provide an objective and reliable method to evaluate axial motor symptoms and quantify the therapy–response to these symptoms.

In the real world, most patients with PD take several kinds of antiparkinsonian drugs at the same time. Among different kinds of antiparkinsonian drugs, DA and dopamine reuptake inhibitors, such as MAOB-I, are the most widely used adjuvant drugs (Gray et al., 2022). In our study, of the 44 patients with PD enrolled in this research, nine patients were treated with levodopa; 15 patients were treated with levodopa and DA; and seven patients were treated with the combination of levodopa, DA, and MAOB-I. After controlling for LED, we found no significant difference among the three groups. Similar results were also revealed in a recent study which demonstrated no difference in patient-reported quality of life improvement between patients receiving DA or MAOB-I, as adjuvant therapy for PD treatment (Gray et al., 2022). In addition, quality-adjusted life years between DA and MAOB-I exhibit no significant difference (McIntosh et al., 2021).

We acknowledge some limitations of the present study. First, we only enrolled 44 PD participants in this research. The small sample size might influence the generalizability of our findings. Second, patients enrolled in our study were not from a *de novo* group. Most of them have taken anti-PD drugs, which might influence their motor performance. Third, freezing of gait, one of the most disabling axial symptoms, was not separately analyzed in this study. The improvement of this symptom under a daily medication regimen needs further study. In addition, the response of axial symptoms to pharmacological therapy cannot be generalized. An effective evaluation of their response to a suprathreshold dose of therapy may be required in patients with axial symptoms.

## Conclusion

Our study demonstrated that axial symptoms were not completely drug-resistant and we found minimal but statistically significant improvements in some kinematic features after the daily medication regimen of patients with PD. The overall changes in axial motor symptom score were associated with the IRs of gait and arising from a chair. The findings presented in our study can help in making tailored, individualized clinical decisions. Further studies, especially of large sample sizes with *de novo* patients, are needed to evaluate the possible influence of different drug combinations on the improvement of motor symptoms.

## Data availability statement

The original contributions presented in the study are included in the article/Supplementary material, further inquiries can be directed to the corresponding author.

## Ethics statement

The studies involving human participants were reviewed and approved by the Ethical Committee of Tongji Hospital. The patients/participants provided their written informed consent to participate in this study.

## Author contributions

LJ designed the research and revised the manuscript critically. QG and LP revised the manuscript. ZW drafted the manuscript. ZW and RH performed the research, collected and analyzed the data, and revised the manuscript. SL, KP, and AL helped in data collection and analysis. YG, YJ, XS, and HZ helped in developing the algorithm system. All authors have read, revised, and approved the final version of the manuscript for publication.
