# Supplementary material for: Technology-based therapy-response evaluation of axial motor symptoms under daily drug regimen of patients with Parkinson’s disease
Source: Front Aging Neurosci. 2022 Aug 5;14:901090. doi: 10.3389/fnagi.2022.901090 (PMC9389404; doi:10.3389/fnagi.2022.901090)
Supplement: Supplementary file 1 [file Table_1.docx]

**Supplementary Table 1. Specific therapy administered during the evaluation of the patients**

|  | **N** | **Morning LED (mg)** | **LEDD (mg)** |
| --- | --- | --- | --- |
| **Levodopa only** | 9 | 111.11±22.05 | 344.44±101.38 |
| **Pramipexole only** | 1 | 75.00±0.00 | 75.00±0.00 |
| **Levodopa + MAOB-I** |  |  |  |
| Levodopa + selegiline | 2 | 175.00±35.36 | 400.00±0.00 |
| Levodopa + rasagiline | 2 | 250.00±35.36 | 587.50±53.03 |
| **Levodopa + DA** |  |  |  |
| Levodopa + piribedil | 4 | 150.00±00.00 | 437.50±25.00 |
| Levodopa + pramipexole | 11 | 139.77±58.05 | 425.00±147.80 |
| **Levodopa + Amantadine** | 1 | 225.00±0.00 | 550.00±0.00 |
| **Levodopa + Amantadine+ Rasagiline** | 1 | 425.00±0.00 | 750.00±0.00 |
| **Levodopa + DA+ MAOB-I** |  |  |  |
| Levodopa + piribedil + selegiline | 1 | 200.00±00.00 | 550.00±00.00 |
| Levodopa + pramipexole + selegiline | 1 | 233.34±00.00 | 783.34±00.00 |
| Levodopa + pramipexole + rasagiline | 5 | 252.50±45.41 | 512.50±154.11 |
| **D+DA+ MAOB-I+ Amantadine** |  |  |  |
| Levodopa + pramipexole+ selegiline + Amantadine | 1 | 425.00±0.00 | 775.00±0.00 |
| Levodopa + pramipexole+ rasagiline + Amantadine | 1 | 387.50±0.00 | 712.50±0.00 |
| **Levodopa + pramipexole + amantadine** | 3 | 220.83±41.25 | 570.83±153.55 |
| **Levodopa + selegiline + pramipexole+ entacapone** | 1 | 275.00±0.00 | 725.00±0.00 |

Data were shown as mean ± standard deviation (SD); LED, levodopa equivalent dose; LEDD, levodopa equivalent daily dose; DA, dopamine agonist; MAOB-I, monoamine oxidase type B inhibitors; NA, not applicable.
